# Associations of lifestyle behaviors with overweight and obesity: a cross-sectional study in Shenzhen, China

**DOI:** 10.3389/fnut.2026.1788311

**Published:** 2026-05-18

**Authors:** Shaojuan Zhao, Leyao Tang, Ni Xiong, Liping Liang, Xin Yin Wu, Chuanning Yu, Xueling Wei, Xuanzhen Wu, Wenjie Dai

**Affiliations:** 1 Clinical Psychology Department, Shenzhen Futian Third People’s Hospital, Shenzhen, Guangdong, China; 2 Department of Epidemiology and Health Statistics, Xiangya School of Public Health, Central South University, Changsha, Hunan, China; 3 Shenzhen Longhua District Center for Chronic Disease Control, Shenzhen, Guangdong, China; 4 The Born in Guangzhou Cohort Study Group, Department of Pediatrics, Guangzhou Women and Children’s Medical Center, Guangzhou Medical University, Guangzhou, Guangdong, China

**Keywords:** China, lifestyle behaviors, obesity, overweight, prevalence

## Abstract

**Background:**

The prevalence of overweight and obesity is increasing, which may lead to adverse health-related outcomes and severe economic burdens. Therefore, early attempts to find the intervention targets to reduce its prevalence are important. This study aimed to identify the association of lifestyle behaviors with overweight and obesity in Shenzhen, China.

**Methods:**

This population-based cross-sectional survey was conducted in Longhua District, Shenzhen. Data on socio-demographic information, lifestyle behaviors, and disease-related characteristics were collected. The body mass index (BMI) value was calculated by using weight in kilograms (kg) divided by the square of height in meters (m^2^). A BMI value of 24.0–28.0 and ≥28.0 kg/m^2^ was considered as overweight and obesity, respectively. Multivariable logistic regression analyses were used to identify the independent associations of lifestyle factors with overweight/obesity. Subgroup and moderation analyses were performed to examine the potential modifying effects of age, sex, marital status, and income level.

**Results:**

A total of 1,429 participants were included in this study. The prevalence of overweight/obesity was 46.5% [95% confidence interval (CI): 43.9–49.1%]. Multivariable analyses showed those who did not consume spicy food daily [adjusted odds ratio (aOR) = 0.76, 95% CI: 0.59–0.96] and those who napped for 1–30 min per day (aOR = 0.64, 95% CI: 0.46–0.88) were at a lower risk of overweight/obesity. Subgroup analyses showed that the association between daily nap duration and overweight/obesity was moderated by sex and marital status.

**Conclusion:**

Almost half of the general adults in Shenzhen were obese or overweight. Daily nap duration and the frequency of spicy food consumption may be served as intervention targets.

## 1 Introduction

The number of people with overweight and obesity is high and expected to increase (1). The worldwide prevalence of obesity was more than doubled between 1990 and 2022, and was predicted to rise to 24% by 2035 (2). In China, the prevalence of overweight/obesity was estimated to be 70.5% in 2030 (3). Accumulating studies have consistently shown that overweight and obesity are major risk factors for a number of chronic diseases, including hypertension, cardiovascular diseases, and musculoskeletal disorders (2, 4, 5). Obesity conferred a 2.60 times higher risk of hypertension and a 3.7 times higher risk of diabetes (6, 7). Furthermore, overweight and obesity pose severe threats to the social economy. For example, it has been estimated that the global costs of overweight/obesity will reach 3 trillion United States Dollars (USD) per year by 2030 and more than 18 trillion USD by 2060 (8), and the medical costs attributable to overweight/obesity will be 417.8 billion Chinese Yuan (CNY) in 2030, accounting for about 22.0% of China’s total medical costs (9). Based on these concerns, enhanced efforts to reduce the prevalence of overweight/obesity are imperative, and early attempts to find the intervention targets are the prerequisite.

Lifestyle behaviors, such as smoking, drinking, physical activity, naps, and eating habits, were modifiable factors which could be served as intervention targets. Their associations with overweight/obesity have been well-established by previous studies (10–13). However, findings differed significantly by study area. For example, Yang et al. (14) found that spicy flavor and spicy food frequency were positively associated with general obesity in Chinese rural populations. However, contradictory results were observed in Chinese adults by Shi et al. (15). Additionally, Alanazi et al. (16) found that obese and overweight individuals were more likely to be current smokers, while in northeast China, current smokers were found to have a lower body mass index (BMI) value than non-smokers (17). In terms of drinking, a previous study based on the United Kingdom Biobank (UKB) database showed no associations between alcohol consumption and adiposity markers for either sex (18), while Song et al. found that drinking was one of the main factors associated with overweight and obesity in northwest China. The inconsistent findings may be explained by the differences in sociocultural environment, economic levels, and population mobility across different study areas (19). As one of China’s special economic zones, Shenzhen is highly developed and characterized by a large floating population. However, no prior studies have explored the associations of lifestyle behaviors with overweight/obesity among general adults in this city. Therefore, the present study aimed to identify such associations in Shenzhen, China by comprehensively assessing whether smoking, drinking, physical activity, nap duration, and dietary habits can be served as intervention targets to reduce the prevalence of overweight/obesity in this area.

## 2 Materials and methods

### 2.1 Study design and participants

This was a population-based cross-sectional study conducted in Longhua District of Shenzhen, China from September 2023 to November 2023. Participants were enrolled using the multi-stage stratified random sampling method. Specifically, four streets namely Guanlan (10 communities), Dalang (12 communities), Longhua (13 communities), and Minzhi (14 communities), were randomly chosen from the six streets in Longhua District. Three communities were then randomly selected from each of these streets. Subsequently, one residential group was randomly selected from each community, and 120 households were randomly selected from each residential group. Finally, one eligible family member from each household was invited to participate in this study. The inclusion criteria were: (1) aged ≥18 years and (2) had lived in Shenzhen for at least 6 months in the past year. Those who were pregnant or unable to participate due to language barriers, physical limitations, or mental problems were excluded. Those with missing values were further excluded from data analyses.

According to the following formula for sample size estimation on prevalence rate: $n=(Z^{2}\times P\times (1-P))/d^{2}$ , a sample size of 595 was obtained based on a two-sided 95% confidence level (*Z* = 1.96), a margin of error of 4% (*d* = 0.04), and an estimated prevalence rate of 45% (20). A minimum required sample size of 1,369 was determined based on a design effect of 2.0 and a non-response rate of 15%.

### 2.2 Data collection

Data collected in this study included socio-demographic characteristics, disease-related characteristics, and lifestyle behaviors by a research team of 16 investigators who received unified training prior to the survey and had at least a Bachelor’s degree in Medicine. Eight well-qualified investigators with at least three times experience in data collection conducted face-to-face interviews to collect information on socio-demographic characteristics and lifestyle behaviors using a well-validated questionnaire. The interview took approximately 15 min. The questionnaire was pre-tested on a small sample of 50 respondents to ensure the respondents understood the questions and that there were no issues with the choice of wording. Disease-related characteristics were collected by anthropometric measurements and laboratory testing.

#### 2.2.1 Anthropometric measurements

Six licensed medical personnel with at least 3 years of work experience conducted anthropometric measurements to collect information on height, weight, and blood pressure. Specifically, height was measured using a non-elastic tape measure with a length of 2.0 m and a minimum scale of 0.1 cm; weight was measured using a G&G TC150KA electronic scale with a minimum graduation of 0.01 kg and a maximum capacity of 150 kg; and blood pressure was measured using an Omron HBP-1300/1320 electronic sphygmomanometer, with readings recorded to the nearest 1 mmHg. All devices were calibrated according to the manufacturer’s instructions prior to the survey. The specific measurement procedures were as follows: participants stood barefoot with their heels together, arms at their sides, and head positioned in the Frankfort horizontal plane. Height was recorded at the end of normal exhalation; weight was measured with participants wearing light indoor clothing and standing upright on the center of the scale platform; and regarding blood pressure, participants rested in a seated position for at least 5 min with their arm supported at heart level.

#### 2.2.2 Laboratory testing

Venous blood samples (5 mL) were collected by two licensed nurses to conduct laboratory testing, including Fasting Blood Glucose and blood lipid levels, using relevant assay kits, and all participants were informed 1 day in advance that they needed to participate on an empty stomach. Medical consumables used in blood sample collection included disposable vacuum blood collection tubes, disposable venous blood collection needles, disposable cotton swabs, medicinal alcohol, tourniquet, blood collection tube labels, and blood collection tube rack. All blood samples were stored at room temperature and tested within an hour upon collection.

### 2.3 Study variables

#### 2.3.1 Study outcome

The outcome of this study was overweight and obesity based on the BMI values calculated by weight in kilograms divided by the square of the height in meters. Specifically, a BMI value of <18.5 kg/m^2^, 18.5–24.0 kg/m^2^, 24.0–28.0 kg/m^2^, and ≥28.0 kg/m^2^ was considered as underweight, normal, overweight, and obesity, respectively (21).

#### 2.3.2 Independent variables

Lifestyle behaviors collected in this study included a history of smoking or not, a history of drinking or not, physical activity status, daily nap duration, number of meals per day, spicy food consumption status, and sugar-sweetened beverage consumption status (10–12). Specifically, smoking was defined as consuming more than one cigarette per day for at least 6 months (22); drinking was defined as consuming any amount of any kind of alcoholic beverage in the past 6 months (23); regular physical activity was defined as performing high-intensity activity for at least 75 min or moderate-intensity activity for at least 150 min per week (24); daily nap duration was defined as length of nap per day; number of meals per day referred to the frequency of consumption of regular meals; spicy food referred to any dish prepared with pungent seasonings; and sugar-sweetened beverage was beverages sweetened with different forms of added sugar.

Socio-demographic characteristics included age, sex, nationality, marital status, and income level. Income level was categorized into low and high with a total annual household income of <250,000 CNY and ≥250,000 CNY, respectively. Disease-related characteristics included the comorbidity of hypertension, diabetes, and dyslipidemia or not (25, 26). Hypertension was defined as systolic blood pressure (SBP) ≥ 140 mmHg or diastolic blood pressure (DBP) ≥ 90 mmHg (27); diabetes was defined as fasting blood glucose (FBS) ≥ 7.0 mmol/L (28); and dyslipidemia was defined as meeting one of the following criteria: total cholesterol (TC) ≥ 5.2 mmol/L, triglycerides (TG) ≥ 1.7 mmol/L, low-density lipoprotein cholesterol (LDL-C) ≥ 3.4 mmol/L, or high-density lipoprotein cholesterol (HDL-C) < 1.0 mmol/L (29).

### 2.4 Ethical approval

This study was conducted in accordance with the Declaration of Helsinki. The protocol was approved by the Ethical Committee of Shenzhen Institute of Chronic Disease Prevention and Control (No. SZCCC-2023-034-01-PJ). Written informed consent was obtained from each participant.

Regarding blood sample collection, participants were informed that they could withdraw from the study at any time, even during the blood draw, if they experienced discomfort or pain from the venipuncture. Participants with overweight/obesity, hypertension, diabetes, or other abnormal laboratory results were referred to local community health service centers for further assessment and management. All participants received their individual laboratory reports within 2 weeks after sample collection. Participants were compensated with a gift worth 50 CNY for their time and transportation expenses. No medical care or services were withheld from participants who declined to participate or withdrew from the study, and all residents remained eligible for standard community health services regardless of their participation status.

### 2.5 Statistical analyses

Continuous variables were presented as mean ± standard deviation (SD), and categorical variables were presented as frequencies (*n*) and percentages (%). Multivariable logistic regression analyses were performed based on two models to identify the independent associations of lifestyle factors with overweight/obesity, and the adjusted odds ratios (aORs) and their 95% confidence intervals (CIs) were calculated to quantify the associations. Specifically, Model 1 was adjusted for socio-demographic characteristics, and Model 2 was further adjusted for disease-related characteristics based on Model 1. Multicollinearity was assessed using the variance inflation factor (VIF), with a VIF value of <10 indicating the absence of severe multicollinearity (30). Subgroup analyses were performed by age, sex, marital status, and income level based on the fully adjusted Model 2. Moderation analyses were further performed by introducing interaction terms between each stratification variable (including age, sex, marital status, and income level) and each associated lifestyle factor identified by multivariable analyses. All statistical analyses were performed using SPSS version 27.0. A two-tailed *p*-value of <0.05 was considered statistically significant.

## 3 Results

### 3.1 Characteristics of the study participants

A total of 1,511 individuals were invited. Among them, 1,429 provided complete data and were finally included, which exceeded the minimum required sample size (1,369), and the response rate was 94.5% (1,429/1,511). The age range of the study participants was 19 to 80 years, with a mean age of 42 ± 12 years. In addition, 727 (50.9%) were males, 1,360 (95.2%) were Han nationality, 380 (26.6%) and 695 (48.6%) had a history of smoking and drinking, respectively, and 685 (47.9%) had regular physical activity (Table 1).

**Table 1:** Characteristics of the study participants.

Variables	Category	Frequency (*n*)	Proportion (%)
Age (years)	18–35	461	32.3
	≥35	968	67.7
Sex	Male	727	50.9
	Female	702	49.1
Nationality	Han	1,360	95.2
	Others	69	4.8
Marital status	Married or cohabiting	1,111	77.7
	Others	318	22.3
Income level	Low	556	38.9
	High	873	61.1
Hypertension	No	1,157	81.0
	Yes	272	19.0
Diabetes	No	1,348	94.3
	Yes	81	5.7
Dyslipidemia	No	561	39.3
	Yes	868	60.7
A history of smoking	No	1,049	73.4
	Yes	380	26.6
A history of drinking	No	734	51.4
	Yes	695	48.6
Regular physical activity	No	744	52.1
	Yes	685	47.9
Daily nap duration (minutes)	0	235	16.5
	1–30	582	40.7
	>30	612	42.8
Number of meals per day	≥3	1,303	91.2
	<3	126	8.8
Frequency of spicy food consumption	Every day	456	31.9
	Not every day	973	68.1
Sugar-sweetened beverage consumption	No	589	41.2
	Yes	840	58.8

### 3.2 Prevalence of overweight, obesity, and overweight/obesity

The mean BMI value of the study participants was 23.95 with a range from 20.36 to 27.54. The prevalence of overweight/obesity was 46.5% (95% CI: 43.9–49.1%).

### 3.3 Multivariable associations of lifestyle behaviors with overweight/obesity

**Figure 1:**
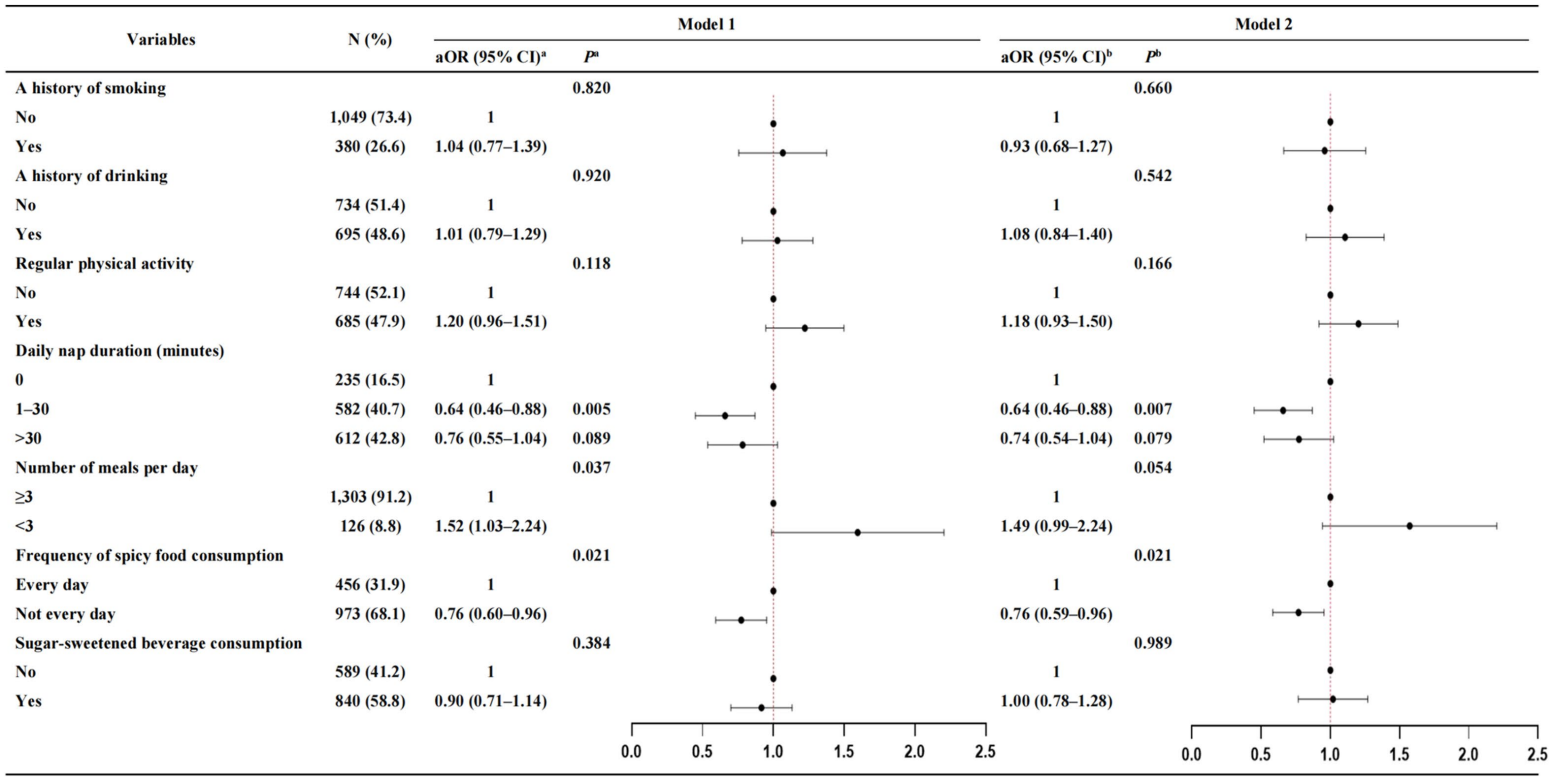
Multivariable associations of lifestyle behaviors with overweight/obesity. ^a^Model 1, adjusted for socio-demographic characteristics; ^b^Model 2, adjusted for socio-demographic and disease-related characteristics; aOR, the adjusted odds ratios; CI, confidence interval.

Multivariable analyses indicated that those who did not consume spicy food daily were at a lower risk of overweight/obesity (aOR = 0.76, 95% CI: 0.60–0.96 in Model 1 and aOR = 0.76, 95% CI: 0.59–0.96 in Model 2), and those who napped for 1–30 min per day were at a lower risk of overweight/obesity (aOR = 0.64, 95% CI: 0.46–0.88 in Model 1 and aOR = 0.64, 95% CI: 0.46–0.88 in Model 2) (Figure 1).

### 3.4 Subgroup analyses

Subgroup analyses showed that among the strata of individuals aged 35 years or above (aOR = 0.68, 95% CI: 0.51–0.92), those who were married or cohabiting (aOR = 0.68, 95% CI: 0.52–0.89), and those with high income (aOR = 0.68, 95% CI: 0.49–0.92), individuals who did not consume spicy food daily were at a lower risk of overweight/obesity; among the strata of females (aOR = 0.52, 95% CI: 0.32–0.85), those who were neither married nor cohabiting (aOR = 0.29, 95% CI: 0.13–0.65), and those with low income (aOR = 0.49, 95% CI: 0.29–0.83), individuals who napped for 1–30 min per day were at a lower risk of overweight/obesity; and among the strata of females (aOR = 0.50, 95% CI: 0.31–0.82), and those who were neither married nor cohabiting (aOR = 0.25, 95% CI: 0.11–0.57), individuals who napped for more than 30 min per day were at a lower risk of overweight/obesity (Supplementary material).

### 3.5 Moderating analyses

Moderation analyses indicated that the association between daily nap duration and overweight/obesity was significantly moderated by both sex (*p* = 0.017) and marital status (*p* = 0.008). Females and those who were neither married nor cohabiting were more sensitive to the protective effects of daily nap duration on overweight/obesity.

## 4 Discussion

To the best of our knowledge, this is the first study to explore the associations of lifestyle behaviors with overweight/obesity among the general adults in Shenzhen, China. The prevalence of overweight/obesity was found to be 46.5% in this area, which was lower than that found in Beijing (51.2%) and higher than that found in Zhejiang, China (42.0%) (31, 32). Besides, the prevalence of overweight/obesity in this study was higher than that in the global population (45.1%) (20). Considering the relationship between overweight/obesity and increased risk of a wide range of chronic diseases, as well as the heavy threats overweight/obesity pose to the economic burden, enhanced efforts to reduce the prevalence of overweight and obesity are imperative in Shenzhen, China.

Consistent with some previous studies (33, 34), this study found that those who did not consume spicy food daily were at a lower risk of overweight/obesity, especially for individuals aged 35 years and above, those who were married or cohabiting, and those with high income. This may be explained by the fact that chili peppers may contribute to increased sugar cravings, and are often consumed with high-calorie foods in China, thereby gaining weight (35). Therefore, spicy food consumption may be served as intervention targets to reduce the prevalence of overweight/obesity, and special attention should be paid to the individual differences in the food consumed together with chili peppers.

Consistent with previous studies (36, 37), this study found that short daily naps can protect against overweight/obesity, particularly among females, those who were neither married nor cohabiting, and those with low income. The underlying mechanism may be that insufficient sleep could lead to decreased leptin levels and increased ghrelin levels, thereby enhancing appetite and food cravings (38). Short naps, serving as a form of “supplemental sleep,” may help restore the hypothalamic regulation of appetite and prevent impulsive eating or high-calorie food intake caused by daytime fatigue. One potential physiological basis for the differential effects of short and long naps lies in their relationship with the sleep cycle. Brief naps typically end before the onset of deep slow-wave sleep, whereas longer naps often terminate during deep slow-wave sleep. However, if awakening occurs during deep slow-wave sleep without completing a normal sleep cycle, it can induce a phenomenon known as sleep inertia—characterized by grogginess, disorientation, and even greater sleepiness than before the nap—which may impair nighttime sleep quality and disrupt circadian rhythms, ultimately increasing the risk of obesity (39, 40).

However, this study also found that for females and individuals who were neither married nor cohabiting, napping for more than 30 min was associated with a lower risk of overweight/obesity, compared to those not napping. A possible explanation is that females experience greater sleep instability due to hormonal fluctuations and are more susceptible to sleep deprivation (41, 42). Similarly, individuals who were neither married nor cohabiting may face more severe nighttime sleep insufficiency (43). Therefore, based on the fact that this study found the association between daily nap duration and overweight/obesity was significantly moderated by both sex and marital status, intervention strategies on daily nap duration to reduce the prevalence of overweight/obesity should take individual differences into consideration. Females and those who were neither married nor cohabiting should be given special concern.

The relationship between smoking and overweight/obesity remained controversial (44–46). Some studies suggested that smoking could suppress appetite, increase the basal metabolic rate, and boost energy expenditure, thereby exerting a certain degree of control over body weight (44, 45), while Carreras-Torres et al. (46) found a positive correlation between smoking and BMI values. Although this study found that a history of smoking was not associated with overweight/obesity, it is still recommended for the general adult population not to smoke due to its adverse effects on a wide range of health-related outcomes (47). Additionally, the relationship between alcohol consumption and overweight/obesity may differ by the indicators used to assess drinking (48). For example, previous studies suggested that mild to moderate drinking was not significantly associated with obesity or even showed a certain protective effect (49, 50), while some studies found that heavy alcohol consumption and alcoholism were related to weight gain and obesity (51). Future studies which comprehensively assess alcohol consumption are still needed.

Consistent with some previous studies (52, 53), this study did not find an association between regular physical activity and overweight/obesity. However, it should still be noted here that regular physical activity plays an important role in managing central obesity (53). In terms of sugar-sweetened beverage consumption, this study did not observe its association with overweight/obesity, which were inconsistent with previous studies (54, 55). This may be attributable to the fact that sugar-sweetened beverage consumption was simply dichotomized as “consumers” versus “non-consumers” in this study, which limited its ability to find a dose–response relationship. In addition to its negative effects on body weight, prior studies have linked sugar-sweetened beverage consumption with an increased risk of diabetes and cardiovascular disease (56). Therefore, it is still recommended for the general population to reduce the intake of sugary beverages on a daily basis.

The strengths of this study included its population-based study design and comprehensive assessment of lifestyle behaviors. However, some limitations still needed to be acknowledged. Firstly, the cross-sectional nature of this study made it difficult to determine any causal relationships between lifestyle behaviors and overweight/obesity. Secondly, recall bias may exist when collecting some information. Thirdly, this study did not consider the adjustment of family history of overweight/obesity. Therefore, future longitudinal studies with further adjustments should be conducted.

## 5 Conclusion

Overweight/obesity was highly prevalent in Shenzhen, China. Daily consumption of spicy food was associated with a higher risk of overweight/obesity, and a short daily nap duration was associated with a lower risk of overweight/obesity. Due to the fact that a cross-sectional study cannot establish causality, it is tentatively suggested for the decision makers to reduce the prevalence of overweight/obesity in Shenzhen, China by promoting healthy lifestyles including consuming less spicy food and taking a short daily nap, especially among females and those who were neither married nor cohabiting. Future longitudinal studies are still needed to confirm the causal relationships.

## Data Availability

The original contributions presented in the study are included in the article/Supplementary material, further inquiries can be directed to the corresponding authors.
