# Supplementary material for: Associations of lifestyle behaviors with overweight and obesity: a cross-sectional study in Shenzhen, China
Source: Front Nutr. 2026 May 18;13:1788311. doi: 10.3389/fnut.2026.1788311 (PMC13222818; doi:10.3389/fnut.2026.1788311)
Supplement: Supplementary file 1 [file Table_1.DOCX]

**Supplementary material**

**1.subgroup analysis-age**


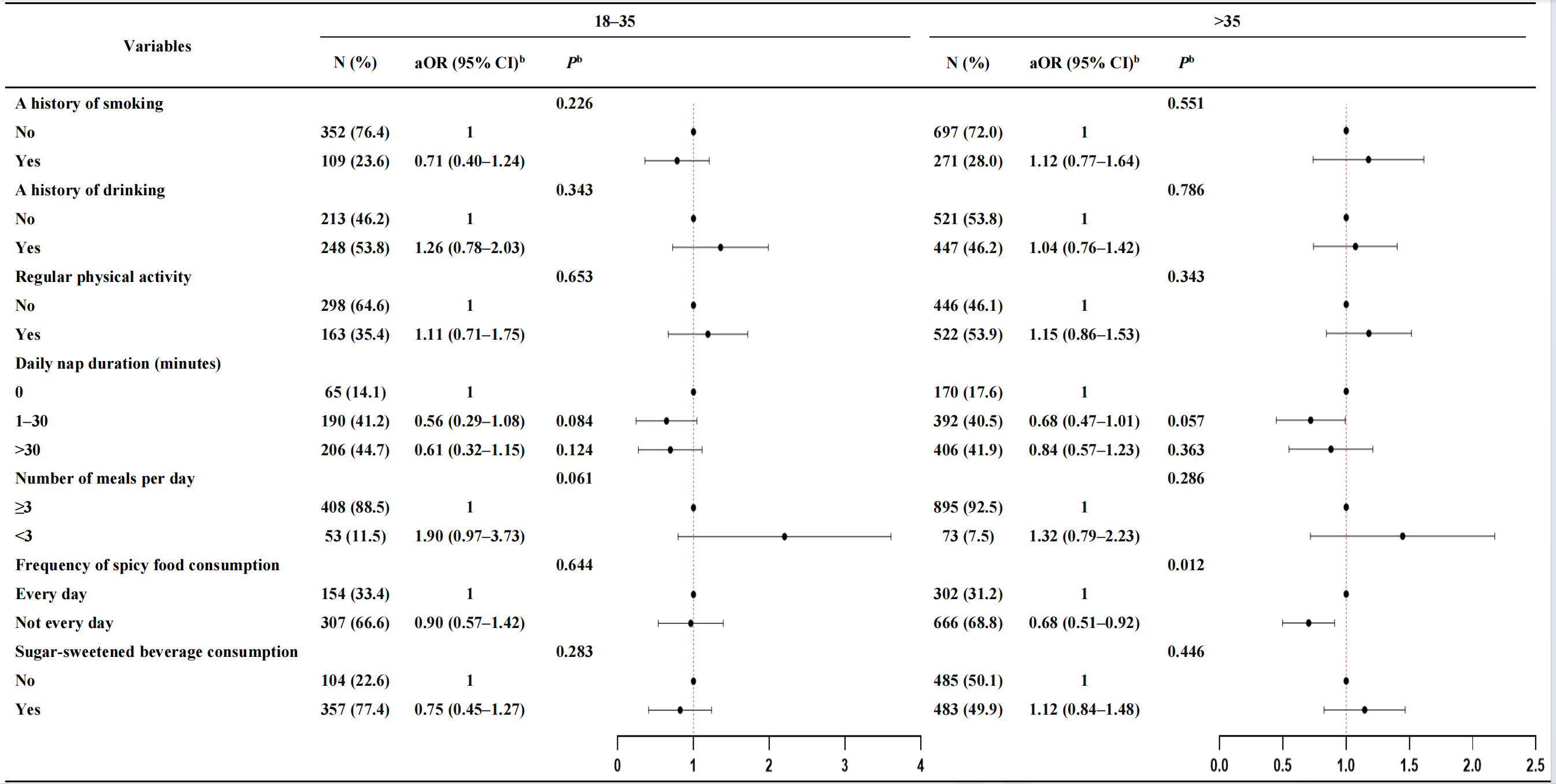


b: Model 2, adjusted for socio-demographic and disease-related characteristics; aOR, the adjusted odds ratios; CI, confidence interval.

**2. Subgroup analysis-sex**


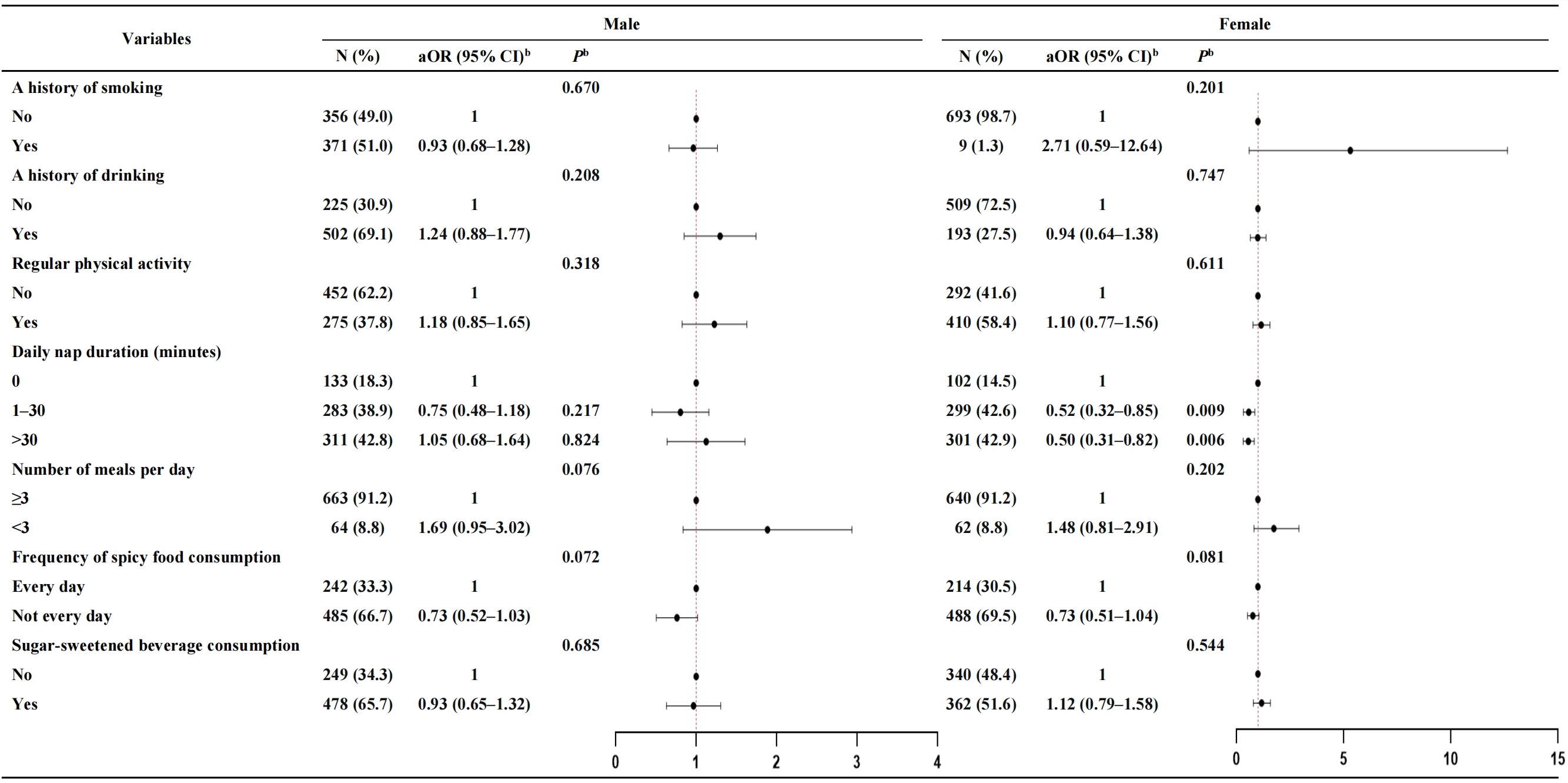


b: Model 2, adjusted for socio-demographic and disease-related characteristics; aOR, the adjusted odds ratios; CI, confidence interval.

**3. Subgroup analysis-marital status**


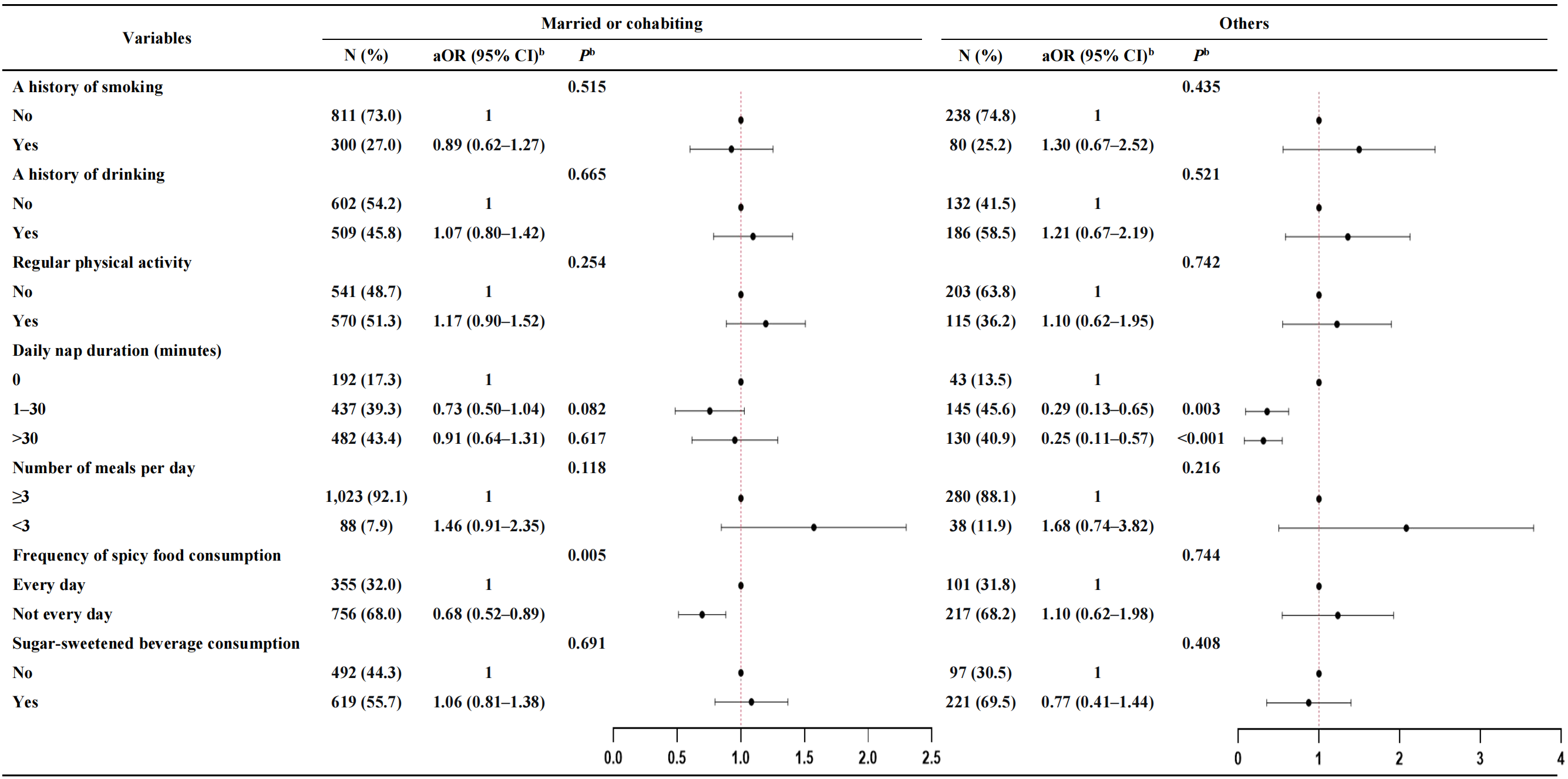


b: Model 2, adjusted for socio-demographic and disease-related characteristics; aOR, the adjusted odds ratios; CI, confidence interval.

**4. Subgroup analysis-income level**


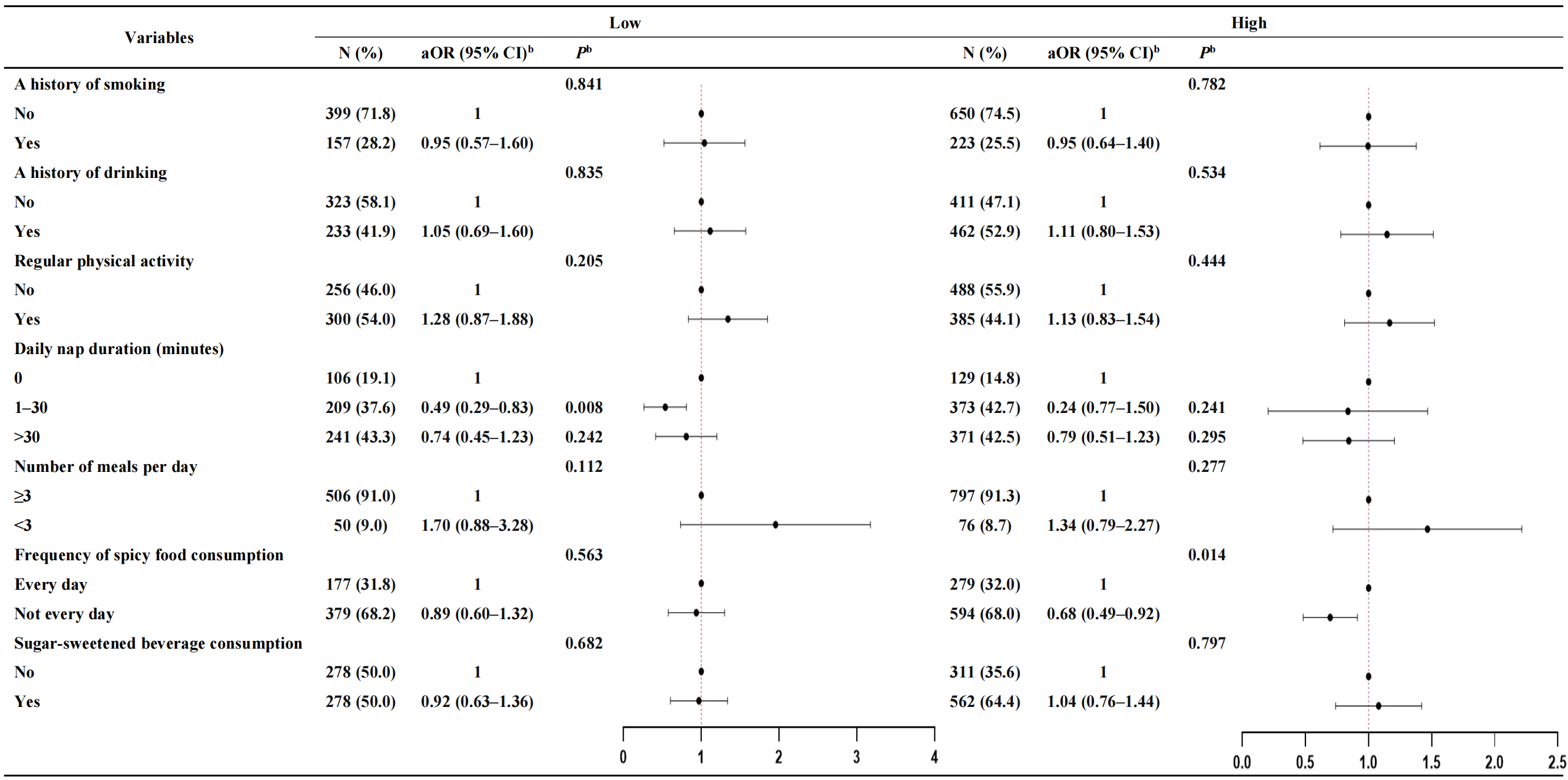


b: Model 2, adjusted for socio-demographic and disease-related characteristics; aOR, the adjusted odds ratios; CI, confidence interval.
